# Exploring Disease Resistance in Pepper (*Capsicum* spp.) Germplasm Collection Using Fluidigm SNP Genotyping

**DOI:** 10.3390/plants13101344

**Published:** 2024-05-13

**Authors:** Nayoung Ro, Gi-An Lee, Ho-Cheol Ko, Hyeonseok Oh, Sukyeung Lee, Mesfin Haile, Jundae Lee

**Affiliations:** 1 National Agrobiodiversity Center, National Institute of Agricultural Sciences, Rural Development Administration, Jeonju 54874, Republic of Korea; nonanona@korea.kr (N.R.); gkntl1@korea.kr (G.-A.L.); hchko@korea.kr (H.-C.K.); zzjiy@korea.kr (H.O.); 2 International Technology Cooperation Center, Rural Development Administration, Jeonju 54875, Republic of Korea; reset00@korea.kr; 3 Department of Horticulture, College of Agriculture and Life Sciences, Jeonbuk National University, Jeonju 54896, Republic of Korea

**Keywords:** *Capsicum* species, disease resistance, Fluidigm genotyping, SNP markers

## Abstract

This study utilized a diverse *Capsicum* accessions (5658) sourced from various species and geographical regions, deposited at the National Agrobiodiversity Center, Genebank. We employed 19 SNP markers through a Fluidigm genotyping system and screened these accessions against eight prevalent diseases of pepper. This study revealed accessions resistant to individual diseases as well as those exhibiting resistance to multiple diseases, including bacterial spot, anthracnose, powdery mildew, *phytophthora* root rot, and potyvirus. The *C. chacoense* accessions were identified as resistant materials against bacterial spot, anthracnose, powdery mildew, and *phytophthora* root rot, underscoring the robust natural defense mechanisms inherent in the wild *Capsicum* species and its potential uses as sources of resistance for breeding. *C. baccatum* species also demonstrated to be a promising source of resistance to major pepper diseases. Generally, disease-resistant germplasm has been identified from various *Capsicum* species. Originating from diverse locations such as Argentina, Bolivia, and the United Kingdom, these accessions consistently demonstrated resistance, indicating the widespread prevalence of disease-resistant traits across varied environments. Additionally, we selected ten pepper accessions based on their resistance to multiple diseases, including CMV, *Phytophthora* root rot, potyviruses, and TSWV, sourced from diverse geographical regions like Hungary, Peru, the United States, and the Netherlands. This comprehensive analysis provides valuable insights into disease resistance in *Capsicum*, crucial for fostering sustainable agricultural practices and advancing crop improvement through breeding strategies.

## 1. Introduction

Pepper, which belongs to the *Capsicum* genus, holds a significant position as one of the world’s most important vegetable crops, including in South Korea [1]. The *Capsicum* genus encompasses around 35 species [2], with 5 of them being cultivated and of economic importance: *Capsicum annuum* L., *Capsicum chinense* Jacq., *Capsicum frutescens* L., *Capsicum baccatum* L., and *Capsicum pubescens* Ruiz and Pav. [3]. However, other species of *Capsicum* species (*Capsicum chacoense* and *Capsicum galapagoense*) have also important traits. According to the data from FAOSTAT [4] spanning from 2010 to 2021, the overall production of pepper has experienced a noteworthy growth of roughly 20.28%. During this period, the production of green peppers increased by 18.12%, while the production of dried peppers exhibited substantial growth at 36.43%. In 2021, global pepper production reached a total of 41.13 million tons, with 36.89 million tons of fresh pepper and 4.84 million tons of dried pepper [4]. In the same year, China led the world in fresh pepper production, producing 16.72 million tons, followed by Turkey with 3.09 million tons and Indonesia with 2.75 million tons. In terms of dried pepper production, India emerged as the foremost producer, contributing 2.05 million tons to the worldwide market [4].

However, the annual production and cultivation of pepper have been impacted by prevalent pepper diseases, resulting in a significant decrease in yield. Notable among these ailments are *Phytophthora* root rot (*Phytophthora capsici*) [5], anthracnose (in the forms of *Colletotrichum scovillei* and *C. truncatum*, previously known as *C. acutatum* and *C. capsici*, respectively [6]), powdery mildew (*Leveillula taurica*) [7], bacterial wilt (*Ralstonia solanacearum*) [8], bacterial spot (*Xanthomonas campestris pv. vesicatora*) [9], cucumber mosaic virus (*CMV*) [10], pepper mild mottle virus (*PMMoV)* [11], tomato spotted wilt virus (*TSWV*) [12], and pepper mottle virus (*PepMoV*) [12,13]. These diseases pose a formidable challenge to control, even with the application of agricultural chemicals.

Molecular markers are widely employed to enhance the effectiveness of plant breeding initiatives, create genetic linkage maps, and identify genes or the quantitative trait locus (QTL) responsible for specific characteristics [14,15,16]. Marker-assisted selection (MAS) and marker-assisted backcrossing (MABC) are key methods in plant breeding, streamlining trait selection. MAS targets specific traits, while MABC hones in on genomic regions in backcross generations [15]. This approach expedites breeding by leveraging codominant markers to detect traits early, eliminating the need for full plant maturity or inoculation, reducing the timeline and generations required compared to traditional phenotypic selection [12,15].Various molecular markers have been developed for selecting resistant pepper varieties against prevalent diseases. Pepper’s bacterial spot resistance genes (Bs2 and Bs3) were cloned [17,18], followed by the development of gene-based codominant markers: 14F/14R for Bs2 and PR-Bs3 for Bs3 [9,18]. Additionally, two dominant markers were reported for detecting a major QTL, Phyto.5.2, associated with resistance to *P. capsici* [19]. Furthermore, codominant markers M3-CAPS and Phyto5NBS1-HRM for the same trait have been developed [5,20]. Two CAPS markers, pvr1-R1 and pvr1-R2, were devised to detect pvr1 and pvr12 alleles for potyvirus resistance in *C. chinense* accessions [21]. Moreover, Pvr4 and Tsw genes were cloned: Pvr4 is a potyvirus resistance gene, originated from *C. annuum* ‘CM334’, while Tsw is a TSWV resistance gene found in *C. chinense* accessions ‘PI159236’ and ‘PI152225’ [22]. Additionally, three SNP markers were developed from the single dominant gene Cmr1, which is associated with CMV resistance [23].

Significant advancements in the field of DNA sequencing and SNP (single-nucleotide polymorphism) genotyping have been achieved in recent decades, including next-generation sequencing (NGS) and high-throughput SNP genotyping [24,25,26,27]. High-throughput SNP genotyping specifically holds substantial promise in the realm of crop breeding [27]. Remarkably, molecular markers can be rapidly developed for SNPs, which are the most common types of genetic variations, exhibiting extensive nucleotide diversity among individual organisms, even within the same species [28]. Presently, a wide array of automated platforms designed for high-throughput analysis have made it possible to process substantial volumes of data rapidly [29,30]. As an illustration, the Fluidigm dynamic arrays employ automated PCR techniques in conjunction with nanofluidic integrated fluid circuits (IFCs) [31]. The Fluidigm platform has found extensive application in the realm of SNP genotyping and the development of SNP markers for distinguishing cultivars in various plant species [32,33,34,35].

Fluidigm SNP-type genotyping markers have been developed for various diseases of pepper, including bacterial spot, anthracnose, *Phytophthora* root rot, powdery mildew, potyviruses, CMV, TMV (tobamovirus), and TSWV [12]. These Fluidigm SNP markers were utilized in the current study. In this research, a large collection of Capsicum germplasm (5658 accessions) from diverse species and geographical locations, preserved within the genebank of the National Agrobiodiversity Center (NAC), Rural Development Administration, was subjected to assessment. We utilized 19 SNP markers through the Fluidigm genotyping system to identify disease-resistant germplasm against eight important diseases of pepper. This geographically and genetically diverse dataset serves as a valuable resource, not only shedding light on the genetic basis of disease resistance in different *Capsicum* species but also offering a nuanced understanding of their distribution and prevalence, thereby informing future breeding strategies and enhancing global crop improvement efforts.

## 2. Results

### 2.1. Summary of Marker Screening Results According to Species

**Figure 1:**
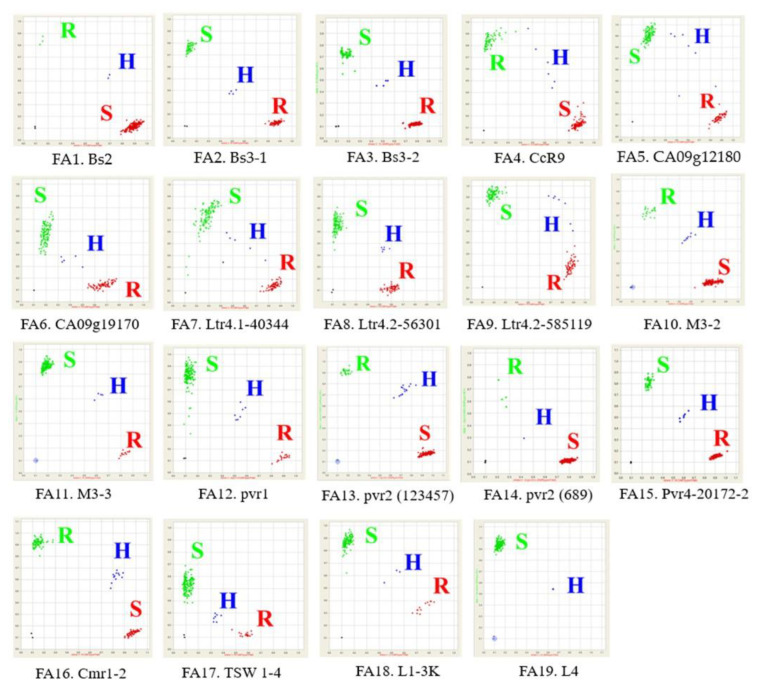
Scatter plots of 19 SNP-type assays for eight diseases, depicting susceptible (S), resistant (R), and heterozygous (H) conditions.

**Figure 2:**
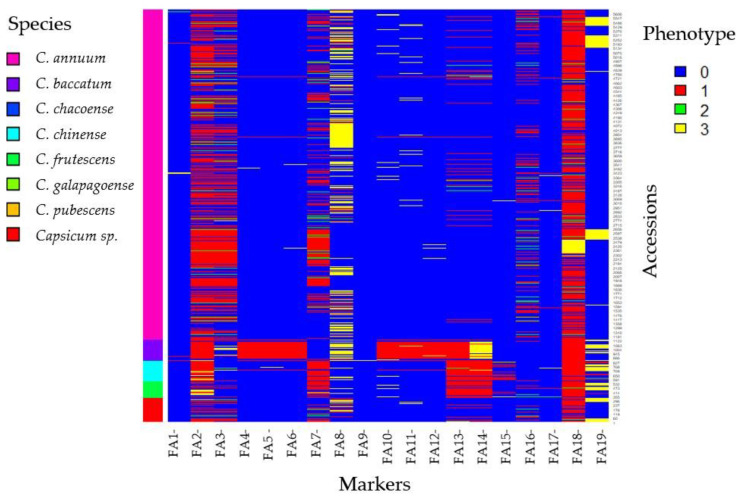
The summary of disease screening results using 19 markers for six diseases using 5658 accessions of *Capsicum* species. Markers name and codes are described in Table 10. Phenotype: susceptible (0), resistant (1), heterozygous (2), and invalid (3).

The summary of the marker screening results according to species involved the analysis of 19 markers utilized for screening resistant accessions against eight diseases: bacterial wilt, anthracnose, powdery mildew, *Phytophthora* root rot, potyvirus, CMV, TSWV, and TMV. This comprehensive analysis involved examining 5658 accessions sourced from various species and diverse geographical locations. Details of the screening results are presented in Table 1, Table 2, Table 3, Table 4 and Table 5, providing a comprehensive breakdown of the number of accessions categorized by species and their corresponding disease reactions for each marker. Additionally, the Fluidigm assay results are visualized in Figure 1 (FA1–FA19), depicting the assay results for each marker. Consolidating all screening results into a single visual representation, showcasing the distribution of accessions and disease reactions across markers and species, a heatmap graph is presented (Figure 2).

#### 2.1.1. Bacterial Spot Resistance

The detailed screening results for resistance to bacterial spot (*X. campestris* pv. vesicatoria) in *Capsicum* accessions, using markers Bs2, Bs3-1, and Bs3-2, are presented in Table 1. This summary count reveals that a total of 22 resistant accessions are identified using marker *Bs2*, 3244 resistant accessions using marker *Bs3-1*, and, using the *Bs3-2* marker, 2166 resistant accessions across all evaluated *Capsicum* species. For *Bs2*, *C. chacoense* emerges as particularly noteworthy, comprising 10 resistant accessions, indicating a substantial proportion of resistance within this species. Other species, such as *C. annuum* (eight), *C. baccatum* (three) and *C. chinense* (one) also exhibit varying counts of resistant accessions. Regarding marker *Bs3-1*, the analysis unveils a distinct pattern of resistance distribution. *C. annuum* stands out with a large count of resistant accessions (2544), followed by *Capsicum* accessions with unknown species (152) and *C. baccatum* (326). Regarding marker Bs3-2, 2013 resistant accessions are identified from *C. annuum*. Additionally, *C. baccatum* (15), *C. frutescens* (20), and other species also exhibit varying counts of resistant accessions.

**Table 1:** Summary of bacterial spot resistance screening in *Capsicum* species using markers.

No.	Species	Bs2			Bs3-1			Bs3-2		
		R	H	S	R	H	S	R	H	S
1	*C. annuum*	8	4	4496	2544	281	1627	2013	303	2182
2	*C. baccatum*	3	0	273	261	0	8	15	3	199
3	*C. chacoense*	10	1	0	9	0	0	0	1	10
4	*C. chinense*	1	0	280	191	1	16	5	2	272
5	*C. frutescens*	0	0	224	84	5	66	20	5	197
6	*C. galapagoense*	0	0	1	1	0	0	1	0	0
7	*C. pubescens*	0	0	2	2	0	0	0	0	2
8	*Capsicum* sp.	0	0	326	152	21	135	112	23	186
Total		22	5	5602	3244	308	1852	2166	337	3048

R: resistant; S: susceptible; and H: heterozygous.

#### 2.1.2. Anthracnose Resistance

The analysis of anthracnose (*C. acutatum*) resistance in various *Capsicum* species, using markers CA09g12180, CA09g19170, and CcR9, reveals consistent results (Table 2). A total of 297 accessions are identified using the CA09g12180 marker, with a similar number identified using the CcR9 marker, while 295 accessions are found to be resistant with the CA09g19170 marker. These three markers consistently show the total counts of resistant and susceptible accessions at both the overall and species levels. Regarding marker CA09g12180, *C. baccatum* emerges as a significant contributor to resistance, boasting the highest count of 248 resistant accessions. Similarly, markers CA09g19170 and CcR9 identify a large number of resistant accessions (244 and 250, respectively) from *C. baccatum*. The consistent resistance across different accessions of *C. baccatum* underscores the robustness of this species in responding to anthracnose. Additionally, *C. annuum* and *C. chacoense* exhibit varying counts of resistant accessions, suggesting their potential as sources of resistance to anthracnose.

**Table 2:** Summary of anthracnose resistance screening in *Capsicum* species using markers.

No.	Species	CA09g12180			CA09g19170			CcR9		
		R	H	S	R	H	S	R	H	S
1	*C. annuum*	23	0	4488	24	0	4480	22	4	4485
2	*C. baccatum*	248	3	24	244	2	23	250	2	23
3	*C. chacoense*	10	1	0	11	0	0	10	1	0
4	*C. chinense*	3	2	277	3	2	270	3	2	276
5	*C. frutescens*	4	1	218	4	1	219	3	1	220
6	*C. galapagoense*	0	0	1	0	0	1	0	0	1
7	*C. pubescens*	0	0	2	0	0	2	0	0	2
8	*Capsicum* sp.	9	1	315	9	0	315	9	0	314
Total		297	8	5325	295	5	5310	297	10	5321

R: resistant; S: susceptible; and H: heterozygous.

#### 2.1.3. Powdery Mildew and Phytophtora Root Rot Resistance

Table 3 presents a comprehensive summary of resistance screening results for powdery mildew and *Phytophthora* root rot in various *Capsicum* species. Three markers, namely Ltr4.1-40344, Ltr4.2-56301, and Ltr4.2-585119, were utilized for powdery mildew resistance, while M3.2 and M3.3 were employed for *Phytophthora* root rot resistance. Distinct patterns emerged across species for powdery mildew resistance. The total number of resistant accessions using these three markers was 299, 291, and 291 for Ltr4.1-40344, Ltr4.2-56301, and Ltr4.2-585119, respectively. Notably, the *C. baccatum* species demonstrated resistance with 248, 243, and 244 accessions using the Ltr4.1-40344, Ltr4.2-56301, and Ltr4.2-585119 markers, respectively. Additionally, *C. annuum* consistently showed resistance, with counts of 22, 21, and 21 accessions for the respective markers, showing its potential as a source of resistance against powdery mildew.

Regarding *Phytophthora* root rot resistance, markers M3.2 and M3.3 revealed interesting results. *C. annuum* stood out with the highest resistance counts of 264 and 254 accessions for the two markers, emphasizing the consistent presence of the resistance gene against phytophthora root rot. Meanwhile, *C. baccatum* accessions displayed a resistant accession count of 258 accessions for M3.2 and 57 accessions for M3.3.

**Table 3:** Summary of powdery mildew and phytophthora root rot resistance screening in *Capsicum* species using markers.

No.	Species	Ltr4.1-40344			Ltr4.2-56301			Ltr4.2-585119			M3-2			M3-3		
		R	H	S	R	H	S	R	H	S	R	H	S	R	H	S
1	*C. annuum*	22	0	4419	21	1	4273	21	3	4469	264	52	4193	254	58	4175
2	*C. baccatum*	248	2	17	243	1	24	244	4	25	258	1	18	57	2	37
3	*C. chacoense*	11	0	0	11	0	0	10	1	0	10	1	0	11	0	0
4	*C. chinense*	5	2	261	3	0	275	3	2	277	260	4	17	259	2	17
5	*C. frutescens*	4	1	211	4	0	220	4	1	219	180	3	41	179	2	41
6	*C. galapagoense*	0	0	1	0	0	1	0	0	1	1	0	0	1	0	0
7	*C. pubescens*	0	0	2	0	0	2	0	0	2	2	0	0	2	0	0
8	*Capsicum* sp.	9	0	310	9	0	307	9	0	316	58	5	261	54	5	260
Total		299	5	5221	291	2	5102	291	11	5309	1033	66	4530	817	69	4530

R: resistant; S: susceptible; and H: heterozygous.

#### 2.1.4. Potyvirus Resistance 

The examination of potyvirus resistance across various *Capsicum* species, utilizing markers pvr1, pvr2-123457, pvr2-689, and Pvr4-20172-2, is presented in Table 4. The cumulative totals emphasize the overall resistance patterns, with a total of 116, 870, 36, and 4231 resistant accessions across pvr1, pvr2-123457, pvr2-689, and Pvr4-20172-2, respectively. Regarding Pvr1, notable resistance is observed in *C. chinense*, where 94 accessions exhibit resistance. *C. frutescens* and *C. annuum* also contribute significantly to the pool of resistant accessions, with 11 and 5 accessions identified as resistant, respectively. Moving on to pvr2-123457, a large number of accessions (779) from *C. annuum*, are identified as resistant, followed by *C. frutescens* and *C. chinense*, with 23 and 11 accessions, respectively (Table 4). For pvr2-689, 26 resistant accessions from *C. annuum* are found to be resistant, followed by 5 accessions from *C. frutescens* and 2 accessions from *C. chinense*. Regarding the Pvr4.20172.2 marker, *C. annuum* (3247 accessions) exhibits the largest count of resistant individuals, followed by *C. chinense* (271 accessions), *C. baccatum* (268 accessions), and *C. frutescens* (195 accessions). Notably, the Pvr4.20172.2 marker, derived from the Pvr4 gene, is present across all species except *C. galapagoense*, indicating its potential for broad-spectrum resistance breeding strategies.

**Table 4:** Summary of potyvirus resistance screening in *Capsicum* species using markers.

No.	Species	pvr1			pvr2-123457			pvr2-689			Pvr4-20172-2		
		R	H	S	R	H	S	R	H	S	R	H	S
1	*C. annuum*	5	5	4499	779	249	3457	26	5	4464	3247	193	872
2	*C. baccatum*	3	0	274	7	0	270	1	0	274	268	1	4
3	*C. chacoense*	0	0	11	0	0	11	0	0	10	10	0	0
4	*C. chinense*	94	11	174	11	1	269	2	0	279	271	0	9
5	*C. frutescens*	11	0	212	23	10	190	5	2	214	195	1	25
6	*C. galapagoense*	0	0	1	0	0	1	0	0	1	0	0	1
7	*C. pubescens*	0	0	2	0	0	2	0	0	2	2	0	0
8	*Capsicum* sp.	3	0	322	50	22	253	2	0	325	238	22	63
Total		116	16	5495	870	282	4453	36	7	5569	4231	217	974

R: resistant; S: susceptible; and H: heterozygous.

#### 2.1.5. CMV, TTSWV, and TMV Resistances

The investigation into disease resistance across various *Capsicum* species reveals nuanced patterns for CMV (marker Cmr1-2), TSWV (marker TSW1-4), and TMV (markers L1-3K and L4) (Table 5). The examination of disease resistance was conducted among various *Capsicum* species against CMV using marker Cmr1-2. A total of 1797 resistant accessions against CMV were identified. A substantial number of resistant accessions (1345 accessions) were from *C. annuum*, followed by *C. chinense* with 245 accessions and C. *frutescens* with 129 accessions. 

In the context of resistance against tomato spotted wilt virus, using marker TSW1-4, *C. chinense* comprised 47 resistant accessions. Additionally, six resistant accretions from *C. annuum* species and also four accessions from *C. frutescens* were found. Generally, a total of 62 resistant accessions were identified for TSWV using the TSW1-4 marker. 

The screening of resistant accessions against tobacco mosaic virus was carried out using two molecular markers, L1-3K and L4. A total of 304 resistant accessions were identified using marker L1-3K, mainly from *C. annuum* with 266 accessions. Additionally, four accessions from *C. frutescens* and three accessions from *C. chinense* were identified as resistant against TMV using the L1-3K marker. Regarding marker L4, only one accession, identified as heterozygous, was found. Considering the marker is from a resistant gene, this one accession from *C. annuum* is predicted to be resistant to TMV.

**Table 5:** Summary of CMV, TSWV, and TMV resistance screening in *Capsicum* species using markers.

No.	Species	Cmr1-2			TSW1-4			L1-3K			L4		
		R	H	S	R	H	S	R	H	S	R	H	S
1	*C. annuum*	1330	272	2905	6	3	4065	251	21	2978	0	1	4510
2	*C. baccatum*	15	2	256	2	0	163	3	0	150	0	0	277
3	*C. chacoense*	0	0	11	0	0	1	0	0	3	0	0	5
4	*C. chinense*	245	2	30	47	8	144	3	0	240	0	0	281
5	*C. frutescens*	129	0	88	4	0	129	4	0	200	0	0	224
6	*C. galapagoense*	1	0	0	0	0	1	0	0	1	0	0	1
7	*C. pubescens*	2	0	0	0	0	2	0	0	2	0	0	2
8	*Capsicum* sp.	75	15	235	3	0	235	43	5	186	0	0	326
Total		1797	291	3525	62	11	4740	304	26	3760	0	1	5626

R: resistant; S: susceptible; and H: heterozygous.

### 2.2. Association of Markers 

The data were summarized in two forms for correlation and cluster analyses: one set includes 15 markers and 4792 accessions, and the other includes 19 markers and 2811 accessions. In both cases, the data consist of complete datasets, indicating that all accessions have valid results for every single marker analyzed. The inclusion of complete datasets enhances the reliability and robustness of the findings derived from the cluster plot analysis, allowing for more accurate interpretations of genetic patterns and disease resistance mechanisms across different marker sets and accession populations.

**Figure 3:**
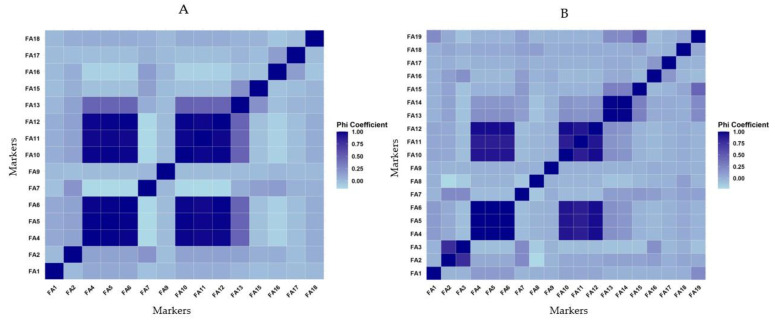
Correlation of 15 markers using 4792 accessions (**A**) and 19 markers using 2811 accessions (**B**) of different Capsicum species. Marker names and codes are presented in Table 10.

A correlation analysis was conducted on markers to assess their similarity and understand the relationship among markers broadly in terms of screening resistant accessions for various diseases by calculating the phi coefficient. The results of the correlation analysis are presented in Figure 3A,B, utilizing different marker combinations and datasets. The findings revealed a strong correlation among the markers, indicating significant similarity in both the count at the species level and the identity of accessions when screening for resistant accessions against anthracnose. Similarly, the markers used for screening powdery mildew showed a strong correlation, suggesting higher similarity in the results. Moreover, the correlation between the markers used for screening anthracnose and powdery mildew was also robust. A large number of accessions were found to be resistant to both diseases, as indicated by the markers used in this study and presented in Figure 3. In general, multiple markers used for screening resistance to a single disease exhibited strong correlations between them.

**Figure 4:**
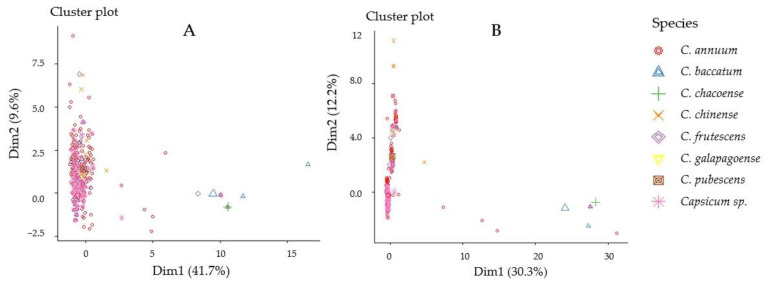
Cluster plots using the datasets of 15 markers included 4792 accessions (**A**) and 19 markers included 2811 accessions (**B**) of Capsicum species after screening out the invalid results.

Cluster plots, represented in Figure 4A,B, visualize the clustering patterns observed in the SNP marker data for Capsicum species. In Figure 4A, where 15 markers and 4792 accessions are analyzed, the first two dimensions of the reduced dimensional space, Dim1 and Dim2, explain 41.7% and 9.6% of the total variance, respectively. In Figure 3B, with 19 markers and 2811 accessions, Dim1 and Dim2 account for 30.3% and 12.2% of the total variance, respectively. Despite the differences in marker count and accession number, both analyses demonstrate substantial variance captured by the first two dimensions. Notably, distinct clusters formed by the *C. baccatum* and *C. chacoense* accessions suggest shared genetic traits linked to resistance against multiple diseases.

### 2.3. Selected Resistant Accession for Multiple Diseases

The examination of disease resistance in various *Capsicum* species, particularly when comparing wild and cultivated varieties, reveal interesting patterns. Accessions with multiple disease resistance are identified (Table 6 and Table 7). Accessions such as IT231144, IT261664, IT283491, IT283493, IT283494, IT283495, and IT283496, showing resistance across bacterial spot, anthracnose, powdery mildew, phytophthora root rot, and potyvirus markers, predominantly belong to the wild species *C. chacoense*. This emphasizes the robust natural defense mechanisms inherent in wild *Capsicum* species, positioning them as crucial genetic resources for breeding programs aimed at bolstering disease resilience. Accessions from *C. chacoense*, originating from diverse locations like Argentina, Bolivia, and the United Kingdom, consistently exhibit resistance. This geographical diversity suggests a widespread prevalence of disease-resistant traits in wild species across different environments. 

Table 7 displays 10 *Capsicum* accessions selected based on their disease resistance to multiple diseases (CMV, *Phytophthora* root rot, potyviruses, and TSWV) using the markers employed for screening. Predominantly, these accessions belong to the *C. chinense* species, but they also belong to other species such as *C. annuum*, *C. baccatum*, and *C. frutescens*. Originating from diverse geographical regions such as Hungary, Peru, the United States, and the Netherlands, these accessions showcase the presence of resistance for multiple diseases within the *Capsicum* genus. These selected materials can serve as invaluable genetic reservoirs for the development of disease-resistant crop varieties. 

### 2.4. Fluidigm Data Compared to Disease Phenotype for CMV and TSWV Viruses

A disease evaluation was conducted for two viral diseases (CMV and TSWV) to compare with the Fluidigm markers developed for screening. Regarding marker Cmr1-2, it demonstrates moderate accuracy in identifying both resistant and susceptible accessions (Table 8). Specifically, it correctly identifies 42.86% of true resistant accessions and 70.22% of true susceptible accessions (Table 8). Notably, the marker exhibits higher accuracy in identifying true susceptible accessions, which is crucial for disease management and breeding programs. However, there are notable inaccuracies, with 29.78% of susceptible accessions incorrectly classified as resistant and 57.14% of resistant accessions incorrectly classified as susceptible, indicating limitations in the marker’s ability to distinguish between resistant and susceptible phenotypes. Considering the total number of accessions tested (2378), the marker Cmr1-2 had an accuracy of 70.14%.

Regarding the TSWV virus, the marker “TSW1-4” shows varying levels of accuracy compared to the CMV marker (Table 8). It correctly identifies 27.27% of true resistant accessions and 99.12% of true susceptible accessions (Table 8). Despite challenges in accurately classifying accessions, particularly in distinguishing between resistant and susceptible phenotypes, the TSWV marker demonstrated high accuracy in identifying true susceptible accessions, which is valuable for disease management and breeding programs. Based on the total number of accessions tested (1820), the marker TSW1-4 had an accuracy of 98.68%.

## 3. Discussion

Peppers (*Capsicum* spp.) are vulnerable to many diseases, which can significantly reduce their yield and quality. To find pepper accessions that are resistant to these diseases, it is crucial to use efficient screening methods in breeding programs. In our study, we used SNP markers with the Fluidigm genotyping system to screen for disease resistance in a large group of pepper plants from the National Agrobiodiversity Center genebank. In this study, various markers from known resistance genes were used to screen for disease resistance. Three dominant resistance genes, Bs1, Bs2, and Bs3, which are not different forms of the same gene (alleles), have been identified as conferring resistance against *X. campestris* pv. vesicatora (Xcv) [36]. The avirulence genes avrBs1, avrBs2, and avrBs3, obtained from Xcv, have been isolated and demonstrated to induce resistances specific to particular races. Notably, the Bs2 gene in pepper (*Capsicum* spp.) exhibits resistance against the most prevalent Xcv races [9]. This study utilized markers designed from the resistance loci of the Bs2 and Bs3 genes to identify bacterial spot-resistant genotypes in pepper. The Bs2 and Bs3 genes are known for conferring resistance to bacterial spot disease caused by *X. campestris* pv. vesicatoria [17,18]. These genes have been identified in certain wild pepper species, such as *C. chacoense* (Bs2) and *C. annuum* (Bs3), but their resistance mechanisms can also be effective in other *Capsicum* species and related plant species. In the previous study, which included a small sample of accessions from various *Capsicum* species, the unique marker BS2 was found to identify resistant accessions exclusively from *C. chacoense* [12]. Our current research expands on this by confirming resistance in the majority of *C. chacoense* accessions (10 out of a total of 11) and also identifying a few resistant accessions from *C. annuum* (8 accessions) and *C. baccatum* (3 accessions) when utilizing markers derived from the Bs2 gene. The larger sample size in our study allowed us to identify resistant accessions not only in *C. chacoense* but also in other species. On the other hand, when employing markers (Bs3-1 and Bs3-3) derived from the resistance locus of the Bs3 gene, a large number of *C. annuum* accessions were identified as resistant to bacterial spot. The variation in resistant accession distributions for bacterial spot using three markers (Bs2, Bs3-1, and Bs3-2) might be due to genetic diversity within the plant population, differences in pathogen strains, and the specific resistance genes from which the markers are derived. According to gene-for-gene interactions between resistance (R) genes and their corresponding avirulence genes, bacterial spot caused by *Xe* has been classified into eleven races (P0–P10) [37]. Bs1 confers resistance against races P0, P2, and P5; Bs2 against races P0, P1, P2, P3, P7, and P8; and Bs3 against races P0, P1, P4, P7, and P9 [37]. Therefore, resistant genes originating from different sources (species) exhibit varied interactions with different pathogen races.

Two important markers (M3-2 and M3-3) derived from a known locus (*Phyto.5.2*) associated with resistance against *Phytophthora* root rot were utilized to screen resistant accessions. The *Phyto.5.2* locus, renowned for its significant impact on *P. capsici* resistance and its ability to confer broad resistance against multiple isolates [38], proved to be a valuable genetic marker for identifying and selecting resistant pepper varieties. These two markers identified resistant accessions from all *Capsicum* species used in this study but predominantly from *C. chinense* and *C. annuum*. A similar report indicated that the dominant OpD04.717 allele, linked to that locus, was present for all *C. chinense* accessions in the authors’ study and for a few *C. annuum* individuals. Accessions of *C. chacoense* were identified as resistant material, which is in line with [39], a study that identified resistant *C. chacoense* accessions. However, the authors of another study [40] reported that they did not identify any resistant genotypes within the *C. chacoense* species. These *C. chacoense* accessions originated from Bolivia, Argentina, the United Kingdom, and Germany, and two accessions were of unknown origin. Regardless of their origin, they were persistently identified as resistant accessions for several diseases, including *Phytophthora* root rot. Resistant accessions of *C. chinense* primarily originated from Hungary, Bolivia, Brazil, Colombia, Costa Rica, Ecuador, and Peru. In contrast, resistant accessions of *C. annuum* mainly came from the United States, Vietnam, South Korea, India, and China. In a previous report, both *C. annuum* and *C. chinense* resistant accessions predominantly originated from Central America and the Caribbean region, where the most durable source of resistance to *P. capsici*, ‘CM334’, was identified [41]. *Capsicum annuum*, *C. chinense*, and to a lesser extent *C. baccatum* have been recognized as sources of resistance against various races of *P. capsici* [42]. In this study, resistant accessions from *C. baccatum* were also identified using both markers. 

Anthracnose resistance is a primary target in chili pepper breeding endeavors. In this study, three SNP markers (*CA09g12180*, *CA09g19170*, and *CcR9*) were employed to screen for anthracnose resistance, yielding consistent results. Resistance was observed across various species of *Capsicum*, suggesting that resistance is not species-specific. However, a notable proportion of resistant accessions were identified from *C. baccatum* compared to other species. This finding aligns with previous research, which has highlighted *C. baccatum* as having higher levels of resistance to anthracnose compared to other *Capsicum* species, making it an essential genetic resource for anthracnose resistance [43,44,45]. Multiple studies have reported sources of anthracnose resistance in pepper from different countries, with *C. baccatum* and *C. chinense* being commonly identified as reservoirs of resistance [46,47,48,49]. However, according to [47], resistant accessions of *C. chinense* are frequently utilized in breeding programs targeting anthracnose resistance due to their genetic proximity to *C. annuum*, facilitating the transfer of resistance genes between species. Notably, no resistant accessions to anthracnose were identified from *C. galapagoense* and *C. pubescens* species.

Powdery mildew resistance has been identified in various species, primarily *Capsicum annuum*, *Capsicum baccatum*, *Capsicum chacoense*, *Capsicum chinense*, and *Capsicum frutescens* (Table 4). Several studies have pinpointed pepper genotypes with varying degrees of resistance against powdery mildew in these species [50,51,52,53,54]. In this investigation, a substantial number of *C. baccatum* accessions are identified as resistant to powdery mildew using three markers: Ltr4.1-40344248 (248 accessions), Ltr4.2-56301 (243 accessions), and Ltr4.2-585119 (244 accessions). These markers yield similar results, indicating the presence of the target resistance gene in *C. baccatum* compared to other species. Notable resistant pepper genotypes include ‘H-V-12’ and ‘4638’ (*C. annuum*), ‘IHR 703’ (*C. frutescens*), and CNPH 36, 38, 50, 52, 279, and 288 (*C. baccatum*) against *L. taurica* [50,51]. However, the number of *C. annuum* accessions resistant to powdery mildew is relatively low (Table 4). Supporting reports suggest that while most *C. annuum* species are susceptible to powdery mildew, *C. baccatum*, *C. chinense*, and *C. frutescens* species often exhibit resistance. This indicates that resistance to powdery mildew is primarily found in *Capsicum* species other than *C. annuum* [51]. Moreover, the dominant pattern of inheritance of powdery mildew resistance in ‘VK515R’, similar to *C. baccatum*, suggests that resistance in ‘VK515R’ may have been introgressed from *C. baccatum*, possibly facilitated by *C. chinense* as a bridge species, given the lack of cross-compatibility between *C. annuum* and *C. baccatum* [55,56,57].

Plant viruses are responsible for considerable reductions in both crop yield and quality on a global scale [58]. Pepper cultivation faces considerable challenges due to the presence of numerous plant pathogens, with over 60 viruses identified as significant threats [59]. Managing these viral pathogens presents difficulties because of their wide range of hosts and the multitude of insect vectors involved. Utilizing resistant cultivars remains the most effective and often the sole approach to mitigating plant viral diseases [60]. The screening of *Capsicum* accessions for resistance to various viral diseases (potyvirus, CMV, TMV, and TSWV) was conducted using SNP markers. Resistance to potyviruses was investigated by using different markers linked to *pvr1*, *pvr2*, and *pvr4* resistant genes [21,61,62]. In this work, we utilized different markers including ones from the resistant genes *pvr1*, *pvr2*, and *Pvr4*. These markers identified resistant accessions predominantly from *C. chinense* (Table 5 and Table 6). *C. chinense* lines emerge as the most promising resource against potyviruses in previous work [39] since the predominant *pvr1* allele protects pepper plants against TEV, PVY (0), and PepMoV [21]. Markers made from the resistant genes *pvr2* and *pvr4* identified resistant accessions primarily from *C. annuum*. Additionally, a few resistant accessions for potyvirus were identified from *C. chinense* and *C. frutescens* using the markers from resistant genes (*pvr2* and *Pvr4*) (Table 5 and Table 6). The dominant locus *Pvr4*, which controls the complete inhibition of viral replication and accumulation, was investigated, and the resistant allele was observed in all but three *C. baccatum*, 56.1% of *C. chinense*, and 12.1% of *C. annuum*, suggesting that those lines carry potential resistance to PVY (0, 1, 2) and PepMoV [39]. On the other hand, accessions from the wild *Capsicum* species (*C. chacoense*), which exhibited resistance to other diseases, were predicted to be susceptible to all viral diseases evaluated in this study. As reported in previous studies [12,58,63], the distribution of resistant accessions across markers derived from the resistant genes pvr1 and pvr2 differed, a trend also observed in our findings. The *pvr1* assay primarily detected resistance alleles in *C. chinense* accessions, while the *pvr2-123457* assay, originating from *C. annuum* and *C. frutescens*, showed a higher frequency of resistant reactions in *C. annuum*. This pattern illustrates the genetic diversity among *Capsicum* species and supports our findings, wherein markers derived from the *Pvr4* gene, originating from *C. annuum*, detected a greater number of resistant accessions compared to markers derived from the *pvr1* gene, which is associated with *C. chinense*. These observations highlight the importance of considering genetic diversity and marker specificity in virus resistance screening within pepper populations. Furthermore, the race specificity of these resistance genes may also contribute to the observed patterns of resistance, underscoring the complex interplay between genetic factors and virus strain specificity in pepper virus resistance.

CMV is one of the most persistent viruses affecting peppers in South Korea [64]. We employed a marker (Cmr1-2) associated with the CMV-resistant gene Cmr1. Over recent decades, diverse sources of resistance to CMV have been uncovered in *Capsicum*. Most of these sources exhibit polygenic resistance controlled by multiple genes. Notable examples include *C. annuum* varieties such as “Perennial” [65,66,67], “Vania” [68], “Sapporo-oonaga”, and “Nanbu-oonaga” [69], as well as *C. frutescens* “BG2814-6” [66], *C. frutescens* “LS1839-2-4”, and *C. baccatum* “PI439381-1-3” [23,69]. In our investigation, resistant accessions were identified from *Capsicum* species other than *C. chacoense* utilized in the study, showing significant variability in terms of accessions. A substantial number of accessions were predominantly identified from *C. annuum* (25%), *C. chinense* (59%), and *C. frutescens* (88%). A probable source of resistance to CMV was discovered in 94.1% of *C. frutescens* and 90.2% of *C. chinense* accessions but not in any genotype from other domesticated or wild species [39]. Additionally, *Capsicum chinense* and *C. frutescens* were also highlighted as good sources of resistance against CMV by [40] Di Dato et al., using the same CAPS marker, as well as through phenotypic assays [66].

We utilized a single marker (TSW1-4) to identify potential resistance among accessions to TSWV, focusing on the Tsw dominant resistant allele. The resistant accessions were predominantly discovered within *C. chinense*, with subsequent findings in *C. annuum*, *C. frutescens*, and *C. baccatum*. These results echo those of a prior study by [39], which documented resistant accessions across a wide spectrum of *Capsicum* species, including *C. chinense*, *C. frutescens*, *C. baccatum*, *C. chacoense*, *C. eximium*, and *C. cardenasii*, alongside a limited number from *C. annuum*. Moreover, previous studies have highlighted the potential for exploiting resistance in *C. annuum* populations from Mexico, Peru, and Spain, which harbor additional alleles that are candidates for resistance [40,70].

Among the recognized genetic factors providing resistance to TMV, the L4 allele at the L locus is known for its wide resistance spectrum against various pathotypes [71]. In our research, we utilized two markers linked to resistance genes, L1 (L1-3K) and L4 (L4). Based on the L1-3k marker, resistant accessions were found in *C. annuum*, *C. chinense*, *C. frutescens*, and *C. baccatum*. However, when considering marker L4, no homozygous accessions were identified; instead, one heterozygous accession was discovered, which we anticipated to be resistant given its association with the resistance gene. In contrast to our findings, a prior study revealed that the dominant resistant allele for the marker (060I2END), linked to L4, was present in nearly all accessions from both domesticated and wild species, except for *C. annuum*, where potential resistance to TMV was observed only in three landraces [39,40]. Moreover, reports have suggested the identification of resistant sources from *C. chacoense* genotypes carrying the L4 allele [11,39].

A comparison of the disease evaluation results and Flufigm SNP genotyping results for CMV and TSWV is essential for validating the efficacy of marker-assisted selection (MAS) in plant breeding programs. Assessing the agreement between traditional disease phenotyping techniques and molecular marker data allows breeders to verify the reliability of SNP markers in identifying resistant and susceptible plant materials. The observed discrepancies between the two methods underscore the intricate nature of disease resistance mechanisms and emphasize the importance of employing complementary approaches in breeding for disease resistance. The high accuracy (98.68%) of the TSWV SNP markers in predicting resistance or susceptibility indicates their potential for expediting breeding efforts and hastening the development of resilient crop varieties. While the accuracy for CMV SNP markers was 70.22%, this analysis provides valuable insights for refining breeding strategies and optimizing marker selection to enhance crop resilience against these devastating viral pathogens.

## 4. Materials and Methods

### 4.1. Plant Materials and Diseases 

In this comprehensive investigation, *Capsicum* germplasm sourced from diverse geographical origins was assessed for the resistances of major pepper diseases, employing the pre-developed Fluidigm SNP markers. The study comprised an extensive collection of 5658 accessions, spanning across seven distinct species (*C. annuum*, *C. baccatum*, *C. chinense*, *C. frutescens*, *C. pubescens*, *C. chacoense*, and *C. galapagoense*). These invaluable genetic resources are conserved at the Genebank of the National Agrobiodiversity Center, Rural Development Administration, the Republic of Korea (http://genebank.rda.go.kr/, accessed on 26 March 2024). 

In this study, a thorough examination was conducted on a diverse collection of 5658 *Capsicum* accessions originating from various regions across the globe. Accessions were categorized based on their species, revealing the dominant presence of *C. annuum*, comprising 80.06% (4530 accessions) of the total. Additionally, other significant species included *C. chinense* (5.0%, 283 accessions), *C. baccatum* (4.91%, 278 accessions), *C. frutescens* (3.98%, 225 accessions), *C. chacoense* (0.19%, 11 accessions), *C. pubescens* (0.05%, 3 accessions), *C. galapagoense* (0.02%, 1 accession), and *Capsicum* sp. (5.78%, 327 accessions). These accessions were sourced from diverse geographical regions, with the majority originating from Asia (47.03%), Europe (23.58%), North America (9.95%), South America (8.59%), Africa (1.19%), and Oceania (0.27%). Additionally, 9.44% of the accessions had an unknown origin. Details of the genetic resource counts are presented in Table 9. Additionally, the *Capsicum* germplasm introduction number (IT), species name, and origins are provided in Supplementary Table S1.

The target diseases of the germplasm screened include eight major pepper diseases: bacterial wilt, anthracnose, powdery mildew, *Phytophthora* root rot, potyvirus, CMV, TSWV, and TMV.

### 4.2. Primer Design for the Fluidigm SNP Type Assays 

The SNP assays used in this study were previously developed by [12]. The primers for SNP-type assays were designed according to specific criteria for the target sequences. These criteria included the following: The target sequences needed to have a length of at least 60 base pairs, encompassing both the region upstream and downstream of the target SNP site but not exceeding 250 base pairs. For SNP assays, only a single SNP could be present within the target sequence. In cases involving insertions or deletions (In/Dels), the length of the In/Del needed to be less than 10 base pairs. Additionally, the G/C content of the target sequence had to be below 65%. A total of 43 primers were created using D3 Assay Design (accessible at https://d3.fluidigm.com/, accessed on 26 March 2024; Fluidigm, South San Francisco, CA, USA), and detailed primer information can be found in Table 10. Each assay comprised three types of primers: a specific target amplification (STA) primer, a locus-specific (LS) primer, and an allele-specific (AS) primer [31]. 

### 4.3. DNA Extraction

Genomic DNA extraction was performed on fresh leaves using the miniprep method outlined in the procedure by [10].The concentration of the extracted DNA was determined using a BioDropµLITE instrument (BioDropUK Ltd., Cambridge, UK) and then adjusted to a final concentration of 50 ng·µL^−1^. Subsequently, this DNA was utilized for conducting SNP-type assays.

### 4.4. Specific Target Amplification 

Prior to conducting the SNP-type assay, a specific target amplification (STA) procedure was employed to enhance the amplification of the amplicon, which included the desired SNP sequences. This step was carried out to increase the likelihood of success in the SNP-type assay, as described in a previous study [31]. Initially, a 10× STA primer pool was assembled, consisting of a mixture containing 2 µL of the STA primer for each of the 24 markers, 2 µL of the LS primer for each of the 24 markers, and 304 µL of DNA suspension buffer supplied by Teknova in Holister, CA, USA.

For each of the 5658 samples, the STA process was conducted using a LightCycler 96 real-time PCR instrument manufactured by Roche in Basel, Switzerland. This procedure was performed in a total reaction volume of 5 µL per sample. The reaction mixture included 2.5 µL of a master mix from Qiagen in Hilden, Germany, 0.5 µL of the 10× STA primer pool, 0.75 µL of PCR-certified water, and 1.25 µL of genomic DNA. The PCR profile consisted of an initial pre-denaturation step lasting 900 s at 95 °C, followed by 14 cycles of a 2-step amplification process, which involved 15 s at 95 °C and 240 s at 60 °C. Subsequently, 3 µL of the amplified product was diluted by mixing it with 97 µL of PCR-certified water before being utilized in the SNP-type assay.

### 4.5. SNP-Type Assay 

To conduct SNP-type assays with the 192.24 IFC, we readied the assay mix and sample mix. Here is an overview of the process: The assay mix consisted of 1.2 µL of PCR-certified water, augmented with 2 µL of 2× assay loading reagent, and enriched by adding 0.8 µL of the assay pre-mix. This pre-mix was composed of 3 µL of each AS primer, 8 µL of each LS primer, and 29 µL of DNA suspension buffer sourced from Teknova in Holister, CA, USA. For the sample pre-mix, we blended 540 µL of 2× Fast Probe Master Mix from Biotium in Fremont, CA, USA, with 54 µL of an SNP-type 20× sample loading reagent, 18 µL of an SNP-type 60× reagent, 6.48 µL of 50× ROX dye obtained from Invitrogen in Waltham, MA, USA, and 11.52 µL of PCR-certified water. In the next step, the sample mix was generated by combining 1.9 µL of each STA product with 2.6 µL of the sample pre-mix in each well of two 96-well plates. Finally, we loaded 3 µL of each sample mix and 3 µL of each assay mix into the 192 sample inlets and 24 assay inlets of the 192.24 IFC, respectively. The SNP-type assays were executed sequentially using three machines: the IFC controller RX, the IFC cycler, and the EP1 system, all provided by Fluidigm in South San Francisco, CA, USA. These procedures were conducted in accordance with the manufacturer’s instructions [31].

### 4.6. Scoring of SNPs 

In each SNP-type assay, two fluorescence signals were examined: FAM (represented on the red Y-axis) and HEX (represented on the green X-axis). Each of these fluorescence signals was associated with specific SNP markers listed in Table 10. The analysis was carried out using Fluidigm SNP genotyping analysis version 4.1.3, developed by Fluidigm in South San Francisco, CA, USA. This software facilitated the identification of three distinct genotypes: “A” and “B” indicated specific homozygous SNP genotypes, while “H” represented a heterozygous SNP genotype, as illustrated in Figure 1.

### 4.7. Disease Evaluation for CMV and TSWV

The disease phenotyping evaluation data were compared with results from the Fluidigm marker, screening to comprehensively assess disease resistance in the tested accessions. Specifically, disease evaluations were conducted to screen for CMV and TSWV in the tested pepper accessions. This integrated approach involved selecting accessions that underwent both disease phenotyping and SNP marker testing, ensuring a thorough evaluation of disease resistance traits. For CMV screening, the methodology outlined by [72] was followed, while for TSWV screening, the protocol described by [73] was adhered to. This comprehensive strategy facilitated comparisons between the disease phenotyping evaluation and SNP marker results, contributing to a better understanding of the diseases. Disease assessments were recorded at 7, 14, and 21 days post inoculation for both diseases. The disease score ranged from 0 to 9, with the following criteria: 1 indicated an incidence rate of less than 1%, 3 indicated an incidence rate exceeding 1% but less than 10%, 5 indicated an incidence rate exceeding 10% but less than 20%, 7 indicated an incidence rate exceeding 20% but less than 50%, and 9 indicated an incidence rate exceeding 50%. A disease score of 0 to 1 was considered resistant, while scores from 3 to 9 were considered susceptible.

### 4.8. Statistical Analysis 

The data were summarized using the Microsoft Excel program. Correlation analysis, clustering, and visualization were conducted using R Studio (version 4.3.2).

**Table 10:** List of Fluidigm SNP assays details.

Fluidigm Assay No.	SNP-Type Assay	Trait	Target Gene or QTL	Position	SNP (Phenotype ^y^)	SNP (Color of Dye ^z^)	Reference
FA1	Bs2	Bacterial spot resistance	*Bs2*	Chr.9	T(R):A(S)	A(R):T(G)	[9]
FA2	Bs3-1	Bacterial spot resistance	*Bs3*	Chr.2	C(R):T(S)	C(R):T(G)	[18]
FA3	Bs3-2	Bacterial spot resistance	*Bs3*	Chr.2	G(R):T(S)	G(R):T(G)	[18]
FA4	CcR9	Anthracnose resistance	*CcR9*	Chr.9	C(R):A(S)	A(R):C(G)	[74,75]
FA5	CA09g12180	Anthracnose resistance	*CcR9*	Chr.9	A(R):C(S)	A(R):C(G)	[74,75]
FA6	CA09g19170	Anthracnose resistance	*CcR9*	Chr.9	C(R):T(S)	C(R):T(G)	[74,75]
FA7	Ltr4.1-40344	Powdery mildew resistance	*Ltr4.1*	Chr.4	AAAAC(R):GAAAT(S)	AAAAC(R):GAAAT(G)	[76]
FA8	Ltr4.2-56301	Powdery mildew resistance	*Ltr4.2*	Chr.4	A(R):C(S)	A(R):C(G)	[76]
FA9	Ltr4.2-585119	Powdery mildew resistance	*Ltr4.2*	Chr.4	C(R):T(S)	C(R):T(G)	[76]
FA10	M3-2	*Phytophthora* root rot resistance	*Phyto.5.2*	Chr.5	T(R):C(S)	C(R):T(G)	[5,20]
FA11	M3-3	*Phytophthora* root rot resistance	*Phyto.5.2*	Chr.5	CAGA(R):GAGT(S)	CAGA(R):GAGT(G)	[5,20]
FA12	pvr1	Potyvirus resistance	*pvr1*	Chr.4	A(pvr1):C(pvr1^+^)	A(R):C(G)	[21,63]
FA13	pvr2-123457	Potyvirus resistance	*pvr2*	Chr.4	A(pvr2^123457^) T(pvr2not ^123457^)	T(R):A(G)	[21,63]
FA14	pvr2(689)	Potyvirus resistance	*pvr2*	Chr.4	A(pvr2-^689^):C(pvr2+^689^)	C(R):A(G)	[21,63]
FA15	Pvr4-20172-2	Potyvirus resistance	*Pvr4*	Chr.10	C(R):G(S)	C(R):G(G)	[21,63]
FA16	Cmr1-2	CMV resistance	*Cmr1*	Chr.2	T(R):G(S)	G(R):T(G)	[23]
FA17	TSW1-4	TSWV	*Tsw1*	Chr.11	TAAACGGAC(R):CAGACGGACCAAAAAAAGGTACGGAC(S)	TAAACGGAC(R):CAGACGGACCAAAAAAAGGTACGGAC(G)	[22]
FA18	L1-3K	TMV resistance	*L1*	Chr.11	C(L1):T(not L1)	C(R):T(G)	[11,77]
FA19	L4	TMV resistance	*L*_4_	Chr.10	A(L_4_):T(not L_4_)	A(R):T(G)	[11,77]

^y^ R: resistant, S: susceptible; ^Z^ R: red (Fam dye), G: green (HEX dye).

## 5. Conclusions

The study utilized a broad range of *Capsicum* genetic resources, totaling 5658 accessions. These accessions were sourced from diverse species and geographic origins, reflecting the rich genetic diversity present within the *Capsicum* genus. While the study primarily focused on screening for resistance against multiple prevalent diseases rather than delving into the mechanisms of disease resistance, this large and diverse collection provides an excellent foundation for identifying valuable genetic resources with potential for future breeding activities. This extensive resource pool will enable breeders to develop new pepper varieties with enhanced disease resistance. Altogether, the use of 5658 *Capsicum* genetic resources highlights the extensive genetic analysis conducted in the study and its significance for future breeding projects aimed at boosting crop resilience and ensuring food security.

## Figures and Tables

**Table 6:** List of selected accessions resistant to multiple diseases (bacterial spot, anthracnose, powdery mildew, *phytophthora* root rot, and potyvirus).

IT	Bacterial Spot		Anthracnose			Powdery Mildew			Phytophthora Root Rot		Potyvirus	Species	Origin
	FA1	FA2	FA4	FA5	FA6	FA10	FA11	FA12	FA13	FA14	FA18		
231144	R	R	R	R	R	R	R	R	R	R	R	*C. baccatum*	United States
261664	R	R	R	R	R	R	R	R	R	R	R	*C. chacoense*	Argentina
283491	R	R	R	R	R	R	R	R	R	R	R	*C. chacoense*	United Kingdom
283493	R	R	R	R	R	R	R	R	R	R	R	*C. chacoense*	Bolivia
283494	R	R	R	R	R	R	R	R	R	R	R	*C. chacoense*	Bolivia
283495	R	R	R	R	R	R	R	R	R	R	R	*C. chacoense*	Bolivia
283496	R	R	R	R	R	R	R	R	R	R	R	*C. chacoense*	Argentina
283501	H	R	R	R	H	R	R	H	H	R	R	*C. chacoense*	Unknown
231145	R	R	R	R	R	R	R	R	R	-	R	*C. baccatum*	Netherlands
261224	R	R	R	R	R	R	R	R	R	-	R	*C. chinense*	Costa Rica
261663	R	R	R	R	R	R	R	R	R	R	-	*C. chacoense*	United Kingdom
283281	R	-	R	R	R	R	R	R	R	R	R	*C. chacoense*	Unknown
283492	R	-	R	R	R	R	R	R	R	R	R	*C. chacoense*	Bolivia

R: resistant; S: susceptible; and H: heterozygous.

**Table 7:** List of selected accessions resistant to multiple diseases (CMV, *phytophthora* root rot, potyvirus, and TSWV).

IT	CMV	Phytophthora Root Rot		Potyvirus	TSWV	Species	Origin
	FA7	FA13	FA14	FA18	FA19		
229195	R	R	R	R	R	*C. chinense*	Hungary
229196	R	R	R	R	R	*C. chinense*	Hungary
229198	R	R	R	R	R	*C. chinense*	Hungary
235726	R	R	R	R	R	*C. chinense*	Peru
235732	R	R	R	R	R	*C. chinense*	United States
235734	R	R	R	R	R	*C. frutescens*	Netherlands
236421	R	R	R	R	R	*C. annuum*	United States
236726	R	R	R	R	R	*C. chinense*	United States
236736	R	R	R	R	R	*C. baccatum*	Peru
229199	R	R	R	R	H	*C. chinense*	Hungary

R: resistant; S: susceptible; and H: heterozygous.

**Table 8:** Comparison of Fluidigm assay results with disease phenotyping evaluation for two viral diseases (CMV and TSWV).

Response	Disease Phenotype	Marker Prediction Count No. of Accession	Marker Accuracy %
True Resistance	7	3	42.86
False Resistance	-	706	-
True Susceptible	2371	1665	70.22
False Susceptible	-	4	-
Overall	n = 2378	n = 2378	70.14
True Resistance	11	3	27.27
False Resistance	-	16	-
True Susceptible	1809	1793	99.12
False Susceptible	-	8	-
Overall	n = 1820	n = 1820	98.68

**Table 9:** The number of *Capsicum* accession counts by species and origins.

No.	Species	Asia	Europe	North America	South America	Africa	Oceania	Unknown	Total
1	*C. annuum*	2477	1134	377	127	49	13	353	4530
2	*C. chinense*	12	69	58	137	4	-	3	283
3	*C. baccatum*	23	31	44	162	2	1	15	278
4	*C. frutescens*	50	24	76	49	6	1	19	225
5	*C. chacoense*	-	4	-	6	-	-	1	11
6	*C. pubescens*	1	-	-	2	-	-	-	3
7	*C. galapagoense*	-	-	1	-	-	-	-	1
8	*Capsicum* sp.	98	72	7	3	4	-	143	327
Total		2661	1334	563	486	65	15	534	5658

## Data Availability

The data are presented in this paper and as a Supplementary File.

## Supplementary Materials

The following supporting information can be downloaded at: https://www.mdpi.com/article/10.3390/plants13101344/s1, Table S1: The *Capsicum* germplasm introduction number (IT), marker screening results, species name, and origins.
